# Veno-occlusive unloading of the heart reduces infarct size in experimental ischemia–reperfusion

**DOI:** 10.1038/s41598-021-84025-y

**Published:** 2021-02-24

**Authors:** Esben Søvsø Szocska Hansen, Tobias Lynge Madsen, Gregory Wood, Asger Granfeldt, Nikolaj Bøgh, Bawer Jalal Tofig, Peter Agger, Jakob Lykke Lindhardt, Christian Bo Poulsen, Hans Erik Bøtker, Won Yong Kim

**Affiliations:** 1 Department of Cardiology, Aarhus University Hospital, Palle Juul-Jensens Boulevard 99, 8200 Aarhus N, Denmark; 2 Department of Clinical Medicine, MR Research Centre, Aarhus University, Palle Juul-Jensens Boulevard, 8200 Aarhus N, Denmark; 3 Department of Intensive Care Medicine, Aarhus University Hospital, Palle Juul-Jensens Boulevard, 8200 Aarhus N, Denmark; 4 Department of Clinical Medicine, Comparative Medicine Lab, Aarhus University, Palle Juul-Jensens Boulevard, 8200 Aarhus N, Denmark

**Keywords:** Cardiology, Myocardial infarction

## Abstract

Mechanical unloading of the left ventricle reduces infarct size after acute myocardial infarction by reducing cardiac work. Left ventricular veno-occlusive unloading reduces cardiac work and may reduce ischemia and reperfusion injury. In a porcine model of myocardial ischemia–reperfusion injury we randomized 18 pigs to either control or veno-occlusive unloading using a balloon engaged from the femoral vein into the inferior caval vein and inflated at onset of ischemia. Evans blue and 2,3,5-triphenyltetrazolium chloride were used to determine the myocardial area at risk and infarct size, respectively. Pressure–volume loops were recorded to calculate cardiac work, left ventricular (LV) volumes and ejection fraction. Veno-occlusive unloading reduced infarct size compared with controls (Unloading 13.9 ± 8.2% versus Control 22.4 ± 6.6%; p = 0.04). Unloading increased myocardial salvage (54.8 ± 23.4% vs 28.5 ± 14.0%; p = 0.02), while the area at risk was similar (28.4 ± 6.7% vs 27.4 ± 5.8%; p = 0.74). LV ejection fraction was preserved in the unloaded group, while the control group showed a reduced LV ejection fraction. Veno-occlusive unloading reduced myocardial infarct size and preserved LV ejection fraction in an experimental acute ischemia–reperfusion model. This proof-of-concept study demonstrated the potential of veno-occlusive unloading as an adjunctive cardioprotective therapy in patients undergoing revascularization for acute myocardial infarction.

## Introduction

Rapid reperfusion by primary percutaneous coronary intervention is the single most important approach to reduce infarct size in patients with acute myocardial infarction^1^. Myocardial infarct size is a major determinant of prognosis. For every 5% increase in infarct size, 1-year all-cause mortality and hospitalizations for heart failure increases by 20%^2^. Although cardioprotective interventions by pharmacological and mechanical conditioning strategies have demonstrated infarct size reduction in experimental as well as proof-of-concept clinical studies, the translation into a clinical benefit has been challenging^3–7^. Hence, continued development of alternative adjunctive cardioprotective therapies to improve outcome following acute myocardial infarction are needed.

Preload, afterload, myocardial contractility, and heart rate are the major determinants of cardiac output^8^. When preload and afterload are reduced, stroke work and thereby myocardial oxygen consumption decrease. Total mechanical unloading of the heart by implantation of a left ventricular (LV) mechanical assist device has been shown to reduce myocardial infarct size in experimental models of ischemia-reperfusion^9–12^. Further, a recent clinical multicenter trial demonstrated feasibility of LV unloading using the ‘Impella CP’ device in anterior ST-elevation myocardial infarction^13^. Insertion of the device caused a 30-min delay until reperfusion, and the trial showed no effect of unloading on mean infarct size after 30 days. In addition, implementation of this invasive approach is challenging in clinical practice. We hypothesized that veno-occlusive unloading of the left ventricle by balloon inflation in the inferior caval vein would be feasible and reduce cardiac work.

In the present study we sought to investigate the cardioprotective potential of veno-occlusive unloading by balloon occlusion of the inferior caval vein in a closed chest porcine model of ischemia–reperfusion injury^14^.

## Results

### Hemodynamic data

One pig in the intervention and one pig in the control group died during reperfusion. Thus, sixteen animals completed the entire study and only results from those 16 pigs are reported.

**Figure 1:**
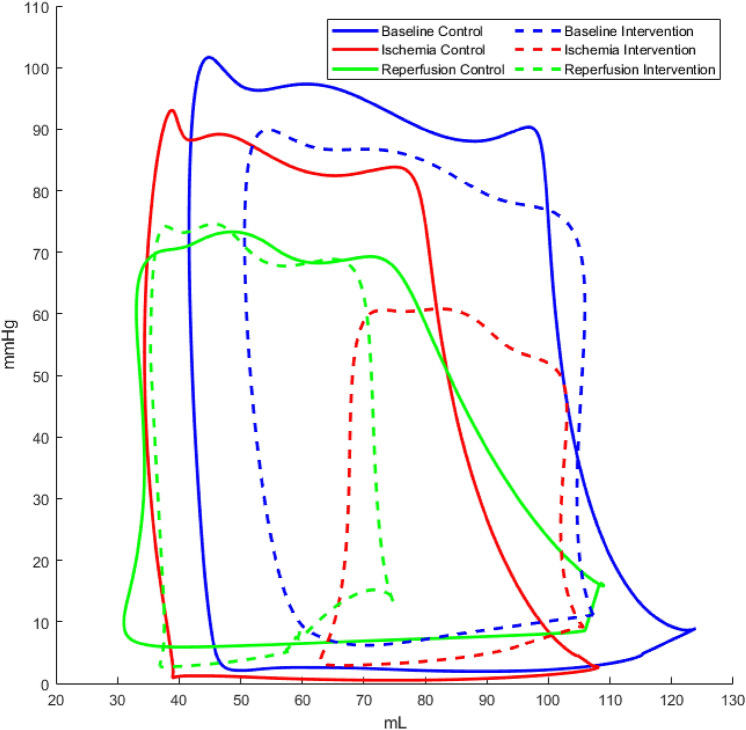
Representative pressure–volume loops from control (solid loops) and intervention (dotted loops) group at baseline, during ischemia, and after reperfusion.

Hemodynamics and body temperature at baseline, during ischemia and after reperfusion in the control versus veno-occlusive unloading group are shown in Table 1. Representative pressure–volume loops are shown in Fig. 1 from control and intervention group at baseline, during ischemia, and after reperfusion.

**Table 1:** Hemodynamics at baseline, after 30 min of ischemia, and after 30 min of reperfusion, by group.

	Control	Baseline vs. ischemia and reperfusion	Intervention	Baseline vs. ischemia and reperfusion	Control vs. intervention
**Heart rate (HR) (beats/min)**					
HR, baseline	60 ± 23		59 ± 13		p = 0.999
HR, ischemia	77 ± 25	p = 0.527	72 ± 35	p = 0.656	p = 0.998
HR, reperfusion	75 ± 25	p = 0.557	92 ± 39	p = 0.142	p = 0.802
**Mean arterial pressure (MAP) (mmHg)**					
MAP, baseline	61 ± 8		65 ± 14		p = 0.932
MAP, ischemia	63 ± 15	p = 0.966	54 ± 16	p = 0.030*	p = 0.718
MAP, reperfusion	53 ± 5	p = 0.045*	54 ± 14	p = 0.136	p = 0.995
**Cardiac output (CO) (L/min)**					
CO, baseline	4.1 ± 2.5		3.6 ± 1.0		p = 0.961
CO, ischemia	3.2 ± 1.1	p = 0.372	3.0 ± 0.9	p = 0.267	p = 0.994
CO, reperfusion	3.0 ± 1.6	p = 0.317	4.0 ± 1.2	p = 0.980	p = 0.943
Stroke work (mmHg * mL)					
Stroke work, baseline	4571 ± 1547		4877 ± 710		p = 0.980
Stroke work, ischemia	3652 ± 1710	p = 0.267	3049 ± 1231	p = 0.010*	p = 0.897
Stroke work, reperfusion	3088 ± 2029	p = 0.155	2683 ± 1418	p = 0.028*	p = 0.985
**Stroke volume (mL)**					
SV, baseline	63 ± 17		60 ± 10		p = 0.995
SV, ischemia	46 ± 25	p = 0.187	49 ± 21	p = 0.436	p = 0.998
SV, reperfusion	45 ± 30	p = 0.256	42 ± 24	p = 0.150	p = 0.999
Cardiac Work per minute (mmHg * mL * HR)					
Cardiac work/min, baseline	295.534 ± 204.316		285.551 ± 61.705		p = 0.999
Cardiac work/min, ischemia	261.824 ± 95.496	p = 0.901	191.097 ± 58.159	p = 0.006*	p = 0.343
Cardiac work/min, reperfusion	206.059 ± 112.412	p = 0.337	245.882 ± 205.180	p = 0.921	p = 0.983
**End diastolic volume (mL)**					
EDV, baseline	123 ± 33		132 ± 16		p = 0.943
EDV, ischemia	104 ± 4	p = 0.428	127 ± 64	p = 0.990	p = 0.900
EDV, reperfusion	98 ± 5	p = 0.346	83 ± 24	p = 0.003*	p = 0.947
**End systolic volume (mL)**					
ESV, baseline	55 ± 19		59 ± 1		p = 0.974
ESV, ischemia	56 ± 13	p = 0.988	61 ± 30	p = 0.997	p = 0.993
ESV, reperfusion	54 ± 25	p = 0.999	42 ± 18	p = 0.174	p = 0.759
**LV ejection fraction (%)**					
LV EF, baseline	56 ± 7		55 ± 10		p = 0.999
LV EF, ischemia	40 ± 19	p = 0.107	49 ± 16	p = 0.279	p = 0.766
LV EF, reperfusion	40 ± 18	p = 0.039*	48 ± 22	p = 0.690	p = 0.905
**Temperature (°C)**					
Temperature, baseline	37.9 ± 0.7		37.6 ± 0.7		p = 0.873
Temperature, ischaemia	37.9 ± 0.7	p = 0.958	38.0 ± 0.5	p = 0.026*	p = 0.999
Temperature, reperfusion	38.2 ± 0.8	p = 0.293	38.1 ± 0.6	p = 0.157	p = 0.999

Comparison between control and intervention group showed no statistically significant differences in the hemodynamic variables or body temperature between the control and intervention group.

**Figure 2:**
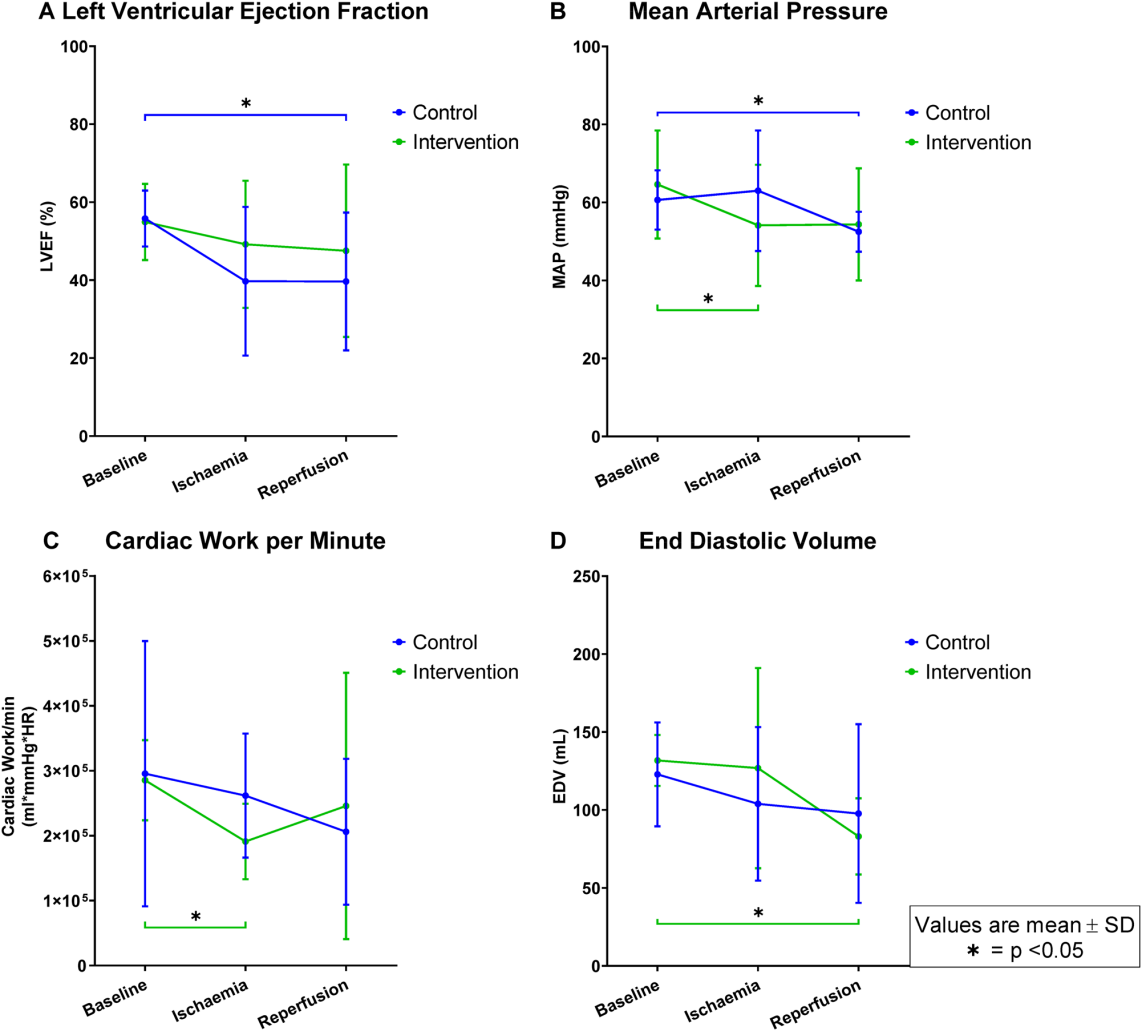
LV ejection fraction, mean arterial pressure, cardiac work per minute and LV end diastolic volume at the same three time points as in Fig. 1 for the control (filled blue circle) and intervention (filled green square) groups.

In the control group, LV ejection fraction was reduced during reperfusion (p = 0.04; Fig. 2A) compared to baseline. The control group also showed a reduction in mean arterial pressure (MAP) during reperfusion compared to baseline (p = 0.045; Fig. 2B).

In the intervention group, stroke work (p = 0.01) and cardiac work (p < 0.01; Fig. 2C) were reduced during ischemia compared to baseline. Only stroke work remained reduced during reperfusion (p = 0.03). Furthermore, the reduction in MAP was significant during ischemia in the intervention group (p = 0.03). LV end-diastolic volumes were reduced during reperfusion in the intervention group compared to baseline (p < 0.01; Fig. 2D). Temperature was significantly increased in the intervention group during ischemia compared to baseline (p = 0.03).

### Infarct size and myocardial salvage

**Figure 3:**
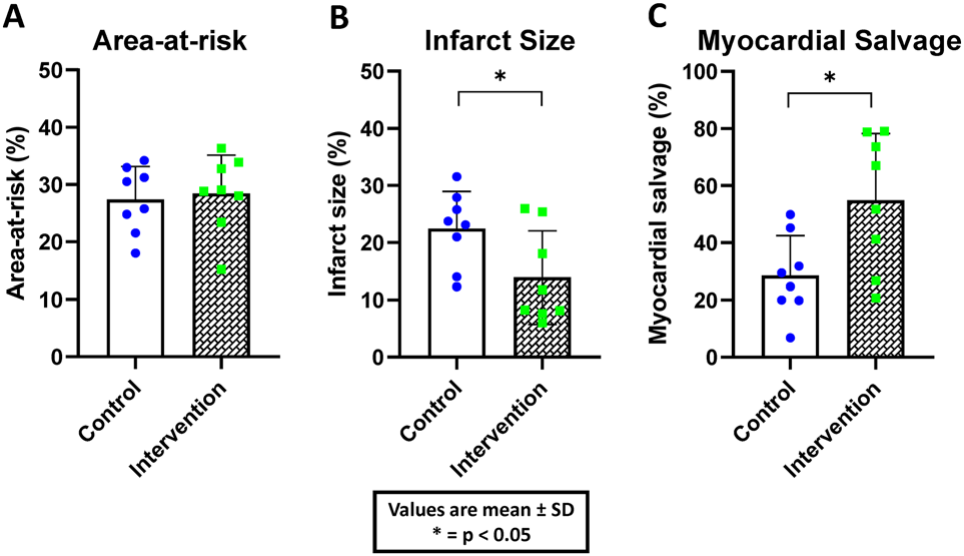
Differences in area at risk, infarct size and myocardial salvage between control and intervention group. The intervention group had significantly smaller infarct size and larger myocardial salvage with similar area at risk. Values are plotted as mean values, error bars represent standard deviation.

The myocardial area at risk was similar in the two groups (Unloading, 28.4 ± 6.7% versus Control, 27.4 ± 5.8%; p = 0.74; Fig. 3A). Unloading significantly reduced infarct size compared with controls (Unloading, 13.9 ± 8.2% versus Control, 22.4 ± 6.6%; p = 0.04; Fig. 3B). Unloading also significantly increased myocardial salvage compared with control (Unloading, 54.8 ± 23.4% versus Control, 28.5 ± 14.0%; P = 0.02; Fig. 3C).

### Correlations between myocardial salvage and hemodynamics

**Figure 4:**
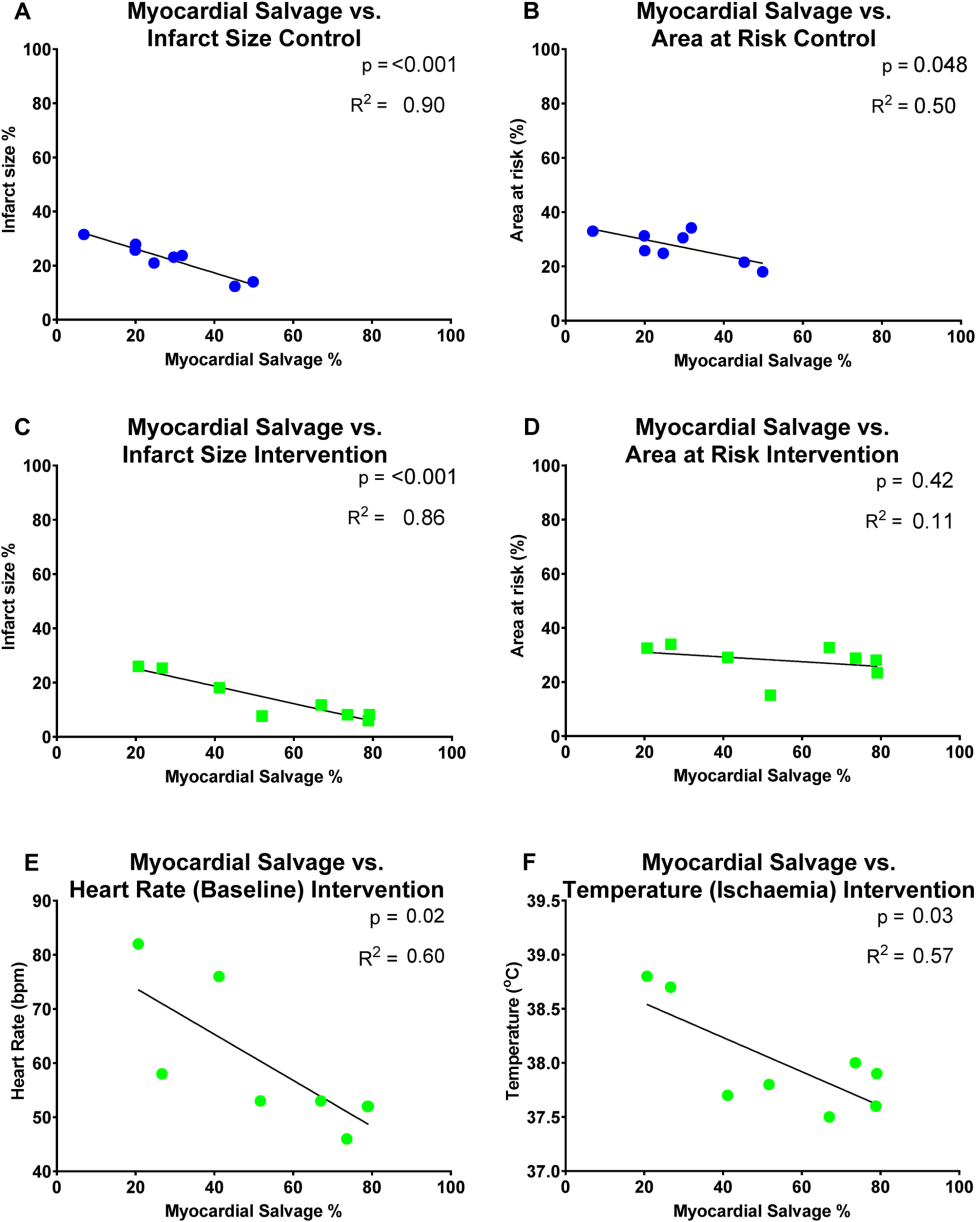
Myocardial salvage correlations with infarct size, area at risk, heart rate and body temperature. See “Results” section for details.

In the control group, myocardial salvage correlated with infarct size (p < 0.001; Fig. 4A) and with the area at risk (p = 0.048; Fig. 4B). Thus, larger myocardial salvage was associated with smaller infarcts and myocardial salvage was reduced with increasing area at risk. In the intervention group, myocardial salvage correlated with infarct size (p < 0.001; Fig. 4C) but not area at risk (p = 0.42; Fig. 4D). Furthermore, myocardial salvage was negatively correlated with heart rate measured at baseline (p = 0.02, Fig. 4E) and temperature measured at baseline (p = 0.046) and during ischemia (p = 0.03, Fig. 4F). Thus, the higher heart rate at baseline and body temperature at baseline and during ischemia, the lower myocardial salvage was observed in the intervention group.

### Correlations between infarct size and area at risk

**Figure 5:**
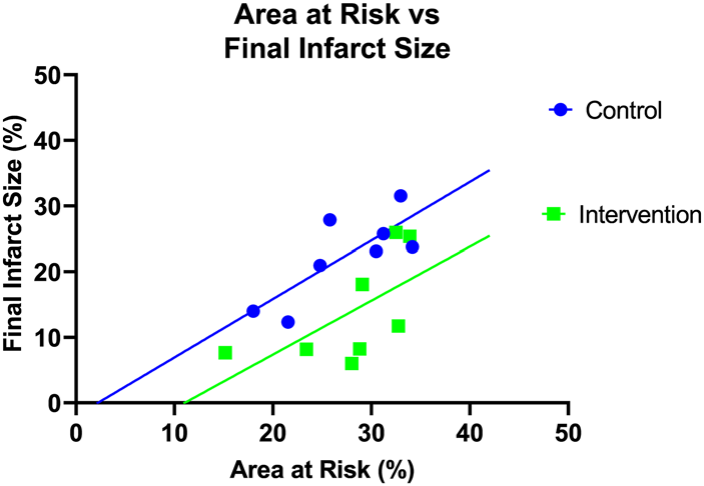
Relation between final infarct size and area at risk for the control (filled blue circle) and intervention (filled green square) groups. See “Results” section for details.

A significant positive correlation was demonstrated between area at risk and final infarct size in the control group (r^2^= 0.62, p = 0.02) while the correlation did not reach statistical significance in the intervention group (r^2^ = 0.38, p = 0.10). The null hypothesis that the two regression lines are identical was rejected with a p-value = 0.03 indicating that for a given area at risk, infarct size was lower with than without veno-occlusion (Fig. 5).

## Discussion

The results of the present study demonstrated that veno-occlusive unloading is cardioprotective in an experimental setting of ischemia–reperfusion injury by reducing myocardial infarct size and increasing myocardial salvage as measured by histopathology. Hemodynamic function improved accordingly.

The mechanistic hypothesis underlying a cardioprotective effect of veno-occlusive unloading against ischemia–reperfusion injury is that unloading reduces cardiac work and myocardial oxygen consumption. We demonstrated that cardiac work was significantly reduced in the intervention group during ischemia compared with baseline. However, we did not show a statistically significant difference in cardiac work per minute during ischemia between the two groups. The reason for this is most likely that ischemia by itself reduces cardiac work. Furthermore, since the variation between animals was evident at baseline, a larger sample size would be needed to show statistical significance. Hence, the best estimate of the reduction in cardiac work per minute due to veno-occlusive unloading was the relative reduction induced by unloading compared to baseline. Unloading not only reduces cardiac work but also reduces wall stress and extravascular coronary compression allowing an increase in collateral blood flow^15^, representing an additional mechanism by which veno-occlusive unloading exerts cardioprotection. To further explore this hypothesis, myocardial perfusion scan or microsphere technique is needed to measure regional myocardial blood flow.

In the present study we did not directly measure myocardial oxygen consumption. Rather, we measured cardiac work from LV pressure volume measurements. We find this justified as it is well documented that external cardiac work is proportionate to myocardial oxygen consumption^16^.

The reduction of cardiac work by veno-occlusive unloading is thought to be mediated through a reduction in both preload, reflected by reduction in end-diastolic volume, and afterload, reflected by reduction in MAP. Thus, while our data confirmed the reduction in MAP during ischemia by unloading, LV end-diastolic volume was reduced at reperfusion but not through ischemia. This finding can be attributed to the fact that left ventricular dilatation is an important pathophysiological mechanism to preserve stroke volume in acute myocardial infarction since for the same amount of myocardial shortening, a dilated left ventricle will generate a higher stroke volume than a non-dilated left ventricle^17^. Accordingly, our data showed that the stroke volume was relatively preserved during ischemia in both groups. Thus, the predominant reduction in cardiac work during ischemia by veno-occlusive unloading was caused by a reduction in afterload, while at reperfusion unloading reduced preload in the intervention group.

Many cardioprotective treatments including ischemic conditioning, pharmacological intervention and mechanical support have been shown to reduce myocardial infarct size in experimental studies of acute ischemia–reperfusion injury. However, translation to clinical benefit for patients suffering from acute myocardial infarction has been challenging^18^. This is generally attributed to the fact that in the clinical setting of acute myocardial infarction many factors are likely to influence outcome^19^. Furthermore, the current reperfusion strategy is already very effective and can reduce the infarct size in the left ventricle to 15–17%^20^. Either multitarget^21^ or combined interventions may be the next step to successfully reduce myocardial ischemia–reperfusion injury in a clinical setting and improve cardiovascular outcome.

Veno-occlusive unloading of the left ventricle by balloon inflation in the inferior caval vein has previously proved efficient and safe in patients undergoing endovascular stenting of the aorta^22^. Possible clinical translation of veno-occlusive unloading by invasive technique depends on future experimental studies to determine whether delayed veno-occlusive un-loading performed shortly before reperfusion will result in cardioprotection. Alternatively, non-invasive approaches to veno-occlusive LV unloading including external occlusion of venous drainage from the legs should be feasible in the clinical setting of acute myocardial infarction^23^ but also positive end expiratory pressure to increase intrathoracic pressure^24^ might be implemented by prehospital personnel. The end diastolic volume has been shown to decrease with both methods, without significantly altering heart rate^23,24^, nor myocardial contractility^25^. Such an approach would allow for unloading already during transport to the hospital and would therefore likely be more efficient than applying unloading at the time of coronary intervention.

Myocardial salvage was correlated to infarct size in both groups. This was expected since myocardial salvage is determined by the area at risk minus infarct size and the area at risk was standardized by occlusion of the left anterior descending artery past the second diagonal. In the control group myocardial salvage was also correlated to the myocardial area at risk while this was not the case for the intervention group, indicating that veno-occlusive unloading has the potential to change this relation. In the intervention group baseline temperature and heart rate were predictive for the myocardial salvage, which indicates that preoperative stress causing increased body temperature and heart rate causes larger infarct size. Furthermore, in the intervention group a significant increase in body temperature was observed during ischemia despite the application of the convective temperature management system that was used to keep the core temperature constant. It appears to have blunted the cardioprotective effects of unloading according to Fig. 4F, showing a negative correlation between temperature and myocardial salvage during ischemia.

There are several limitations to the study. Firstly, we performed the entire ischemia–reperfusion experiments under anesthesia. In real life depending on the hemodynamics it may not be possible and beneficial to reduce stroke work by as much as 25%. The results may, therefore, differ from those in conscious patients. Secondly, the study was conducted as an acute experiment. Hence, we cannot evaluate the effect of unloading on LV remodeling. Lastly, we initiated the unloading from the start of ischemia and maintained it during the entire reperfusion phase. The relative importance of applying unloading during ischemia or after reperfusion was not systematically investigated. We anticipate that delaying the start of unloading will attenuate its impact on infarct size, because we expect that the cardioprotective effect of veno-occlusive unloading occurs primarily during ischemia and very early reperfusion, similar to remote ischemic conditioning^26^ and intravenous metoprolol^27^. There have been conflicting results regarding the timing of mechanical unloading relative to timing of reperfusion. Thus, it has been demonstrated in an experimental study that simultaneous reperfusion and LV mechanical unloading yielded the smallest infarct size compared with unloading during ischemia with delayed reperfusion^28^. Further studies are needed to identify the optimal timing and duration of unloading as well as the optimal cardiac work reductions needed to reduce ischemia–reperfusion injury.

In summary, veno-occlusive LV unloading applied during acute myocardial ischemia–reperfusion reduced myocardial infarct size from 22% of the left ventricle in the control group to 14% in the intervention group. This proof-of-concept study has demonstrated the potential of veno-occlusive unloading as a possible future therapeutic option in the treatment of acute myocardial infarction.

## Methods

### Experimental protocol

We included 18 Danish domestic pigs of female gender weighing 40 kg. All pigs were sedated with intramuscular tiletamine (2.5 mg/kg) and zolazepam (2.5 mg/10 kg) before transport to the surgical facilities. On arrival at the surgical facilities, the pigs were anaesthetized with an initial dose of intravenous propofol (3.3 mg/kg), intubated and mechanically ventilated using a 60% O_2_-air mix. Anesthesia was maintained with intravenous administration of propofol (4 mg/kg/h), and fentanyl (12.5 µg/kg/h). To keep the core temperature constant between 37 and 38 °C during the entire experiment a convective temperature management system was used (Bair Hugger™, 3 M Health Care, MN, USA). We used ultrasound-guided modified Seldinger-technique to position sheaths in the femoral artery and vein for arterial blood pressure monitoring and blood sampling, respectively. Sheaths were introduced into the common carotid arteries and common jugular vein for percutaneous coronary intervention, LV pressure–volume measurements and introduction of a Swan-Ganz catheter. Prior to intravascular instrumentation, 10.000 IU heparin and 300 mg amiodarone were administered intravenously. Real-time pressure–volume loops were obtained using the ADVantage™ system (Transonic SciSense, Elsloo, The Netherlands) with an admittance catheter^29^. The cardiac output was calibrated by thermodilution using the Swan-Ganz catheter. Data were continuously recorded using a multichannel acquisition system and Labchart software (ADInstruments, Oxford, UK).

Veno-occlusive unloading was done using a balloon engaged from the femoral vein into the inferior caval vein just below the right atrium. The unloading was calibrated by temporarily inflating the balloon with X-ray contrast to reach an approximately 25% reduction in stroke work according to the on-line pressure volume measurements. The veno-occlusive calibration procedure was done in all pigs. The pigs were subsequently randomized to control or unloading. Afterwards, X-ray coronary angiography was performed and a 2.0–3.0 mm wide angioplasty balloon (Trek, Abbot Laboratories, IL, USA) was placed in the left anterior descending artery (LAD) past the second diagonal branch and inflated for 60 min. Only in those pigs randomized to unloading, the veno-occlusive unloading, as determined from the calibration procedure, was initiated immediately after occlusion of the LAD in the intervention group and maintained during reperfusion. Invasive blood pressures, heart rate, blood oximetry and end-tidal CO_2_ were continuously recorded.

After four hours of reperfusion, a midline sternotomy was performed and the LAD was sutured at the site of the previous occlusion according to visual inspection of side branches and guided by prior obtained X-ray images. To determine the myocardial area at risk, 10% Evans Blue dye was then injected directly into the left ventricle and allowed to circulate for 30 s. Then ventricular fibrillation was induced using a 9-V DC battery and the heart was harvested, sectioned into 8 mm cross-sections and photographed to determine myocardial area at risk. The slices were then immersion stained with 1% of 2,3,5-triphenyltetrazolium chloride (TTC) (Sigma-Aldrich, Brøndby, Denmark) for 5 min and photographed again to determine the infarct size^30^. A scale for reference was shown for calibration.

The experimental protocol was approved by The Animal Experiments Inspectorate in Denmark (J.nr. 2014-15-2934-01013). The study was carried out in compliance with the ARRIVE guidelines^31^. All methods were carried out in accordance with relevant guidelines and regulations according to current European legislation (Directive 2010/63/EU).

### Data analysis

#### Myocardial tissue staining

The digitized myocardial tissue images were processed using the open-source GNU Image Manipulation Program version 2.10.12. The area at risk was measured from the Evans Blue staining for each slice and the infarct size was measured from the TTC staining. Myocardial salvage index was then calculated as: ((area at risk − infarct area)/area at risk) × 100. The analysis was performed by two independent researchers blinded to the treatment status and the average of the two observers’ measurements was used.

#### Analysis of pressure–volume data

The following measurements were obtained from the pressure–volume data: stroke work, cardiac work per minute calculated as stroke work times heart rate, stroke volume, LV end-diastolic and end-systolic volumes, LV ejection fraction and cardiac output. These measurements were recorded by selecting 10 cardiac cycles at three different time points: at baseline, after 30 min of ischemia, and 30 min into reperfusion. Data were recorded and analyzed by two independent researchers on separate occasions. The mean of the two analyses provided the final result.

### Statistical analysis

A required sample size (n = 16) was calculated to detect an increase in myocardial salvage index ≥ 25% by unloading with 95% power. This predicted reduction was based on the results from a previous study showing the effect of mechanical unloading on myocardial salvage^10^. An experimental failure rate of 10% was expected, so 18 experiments were performed in total. Results are presented as mean ± SD. Residuals were evaluated and determined to be approximately normally distributed. The infarct size, myocardial salvage index and area at risk were compared between the control and intervention group using an unpaired t-test. The hemodynamic variables derived from the pressure–volume measurements were compared using repeated measurement two-way ANOVA by groups (control versus intervention) and according to time points (baseline, ischemia and reperfusion). Tukey post-hoc testing was used to correct for multiple comparisons.

Myocardial salvage correlations and the relation between final infarct size and area at risk were investigated by linear regression analysis. Statistical analysis of the data was performed using GraphPad Prism version 8.2.1. A p-value of < 0.05 was considered significant.

## Data Availability

The datasets generated during and analysed during the current study are available from the corresponding author on reasonable request.

## Abbreviations

LV: Left ventricular
LAD: Left anterior descending coronary artery
TTC: 2,3,5-Triphenyltetrazolium chloride
MA: Mean arterial pressure
